# Performance testing protocol for closed-system transfer devices used during pharmacy compounding and administration of hazardous drugs

**DOI:** 10.1371/journal.pone.0205263

**Published:** 2018-10-31

**Authors:** Alan-Shaun Wilkinson, Michael Charles Allwood, Colin Patrick Morris, Andrew Wallace, Rebecca Finnis, Ewelina Kaminska, Donata Stonkute, Maja Szramowska, Joe Miller, Ian Pengelly, Michael Hemingway

**Affiliations:** 1 Biopharma Stability Testing Laboratory Ltd, BioCity Nottingham, Nottingham, United Kingdom; 2 The Health and Safety Laboratory (HSL), Harpur Hill, Buxton, United Kingdom; CNRS, University Paris-Sud, University Paris-Saclay, FRANCE

## Abstract

**Objectives:**

The United States National Institute for Occupational Safety and Health (NIOSH) is developing a protocol to assess the containment performance of closed system transfer devices (CSTDs) when used for drug preparation (task 1) and administration (task 2) and published a draft protocol in September 2016. Nine possible surrogates were proposed by NIOSH for use in the testing. The objectives of this study were to: (A) select the most appropriate surrogate; (B) validate the NIOSH protocol using this surrogate; and (C) determine the containment performance of four commercial CSTDs as compared with an open system of needle and syringe using the validated NIOSH protocol.

**Methods:**

2-Phenoxyethanol (2-POE) was selected as a surrogate based on its water solubility, Henry’s volatility constant, detectability by mass spectrometry, and non-toxicity. Standard analytical validation methods including system suitability, limit of detection (LOD), and limit of quantitation (LOQ) as well as system cleaning validation were performed. The amount of 2-POE released when the CSTDs were manipulated according to two tasks defined by NIOSH was determined using mass spectrometry coupled to thermal desorption and gas chromatography. This approach allows sensitivity of detection below 1 part per billion (ppb). Equashield, Tevadaptor (OnGuard), PhaSeal, and ChemoClave were assessed according to manufacturers’ instructions for use.

**Results:**

2-POE was tested and validated for suitability of use within the NIOSH protocol. A simple and efficient cleaning protocol achieved consistently low background values, with an average value, based on 85 measurements, of 0.12 ppb with a 95% confidence interval (CI) of ±0.16 ppb. This gives an LOD for the tests of 0.35 ppb and an LOQ of 0.88 ppb. The Equashield, Tevadaptor (OnGuard), and PhaSeal devices all showed average releases, based on 10 measurements from five tests, that were less than the LOQ (i.e. < 0.88 ppb), while the ChemoClave Vial Shield with Spinning Spiros showed average releases of 2.9±2.3 ppb and 7.5±17.9 ppb for NIOSH tasks 1 and 2 respectively at the 95% confidence level. The open system of needle and syringe showed releases, based on two measurements from a single test, of 4.2±2.2 ppb and 5.1±1.7 ppb for NIOSH tasks 1 and 2 respectively at the 95% confidence level.

**Conclusions:**

2-POE proved to be an ideal surrogate for testing of CSTDs using the NIOSH protocol. We propose that a CSTD can be qualified using the NIOSH testing approach if the experimental LOQ is less than 1 ppb and the release values are below the LOQ. Equashield, Tevadaptor (OnGuard), and PhaSeal meet these acceptance criteria and can therefore all be qualified as CSTDs, but the ChemoClave system does not and so would not qualify as a CSTD.

## Introduction

### Background

Many anti-cancer drugs are potentially therapeutic to patients, but unintended exposure to these agents poses an occupational hazard to healthcare workers [1,2]. Healthcare workers exposed to anti-cancer drugs may experience acute symptoms including headache, allergic reactions, and nausea and vomiting, or long-term effects such as genotoxicity, infertility, and fetal abnormalities [2]. These risks prompted the publication of guidelines in the United States (US) by NIOSH and internationally by the Health and Safety Executive (HSE) of the UK for the safe handling of cytotoxic and other hazardous drugs [2–6]. Use of primary engineering controls such as a ventilated cabinet or isolator and personal protective equipment (PPE) are mandated for drug preparation, but the use of a closed system transfer device (CSTD) [1] is currently only recommended as an additional hierarchical layer of operator protection. Without a CSTD, however, the operator is completely reliant upon PPE in the event of a failure of the engineering controls or when accessing the cabinet or isolator to transfer materials in or out. A CSTD can help to further reduce occupational exposure in these scenarios as well as on the ward where cabinets are not available and staff are completely reliant upon the use of PPE [2].

According to NIOSH, CSTDs are drug transfer devices that mechanically prevent the transfer of environmental contaminants into the system and the escape of hazardous drug or vapor concentrations outside the system [1]. CSTDs reduce the risk of needle-stick injuries and the formation of aerosols during the compounding and administration processes, which are common sources of contamination [3,7]. CSTDs help to reduce exposure by containing any aerosol, liquid, or vapor releases. This is achieved by employing either a physical barrier in a closed system that prevents any transfer across the system boundary, or by use of air filtration technology that specifically cleans the air that passes between the environment and the hazardous drug vial [1,8]. Using either system, the operator is protected from exposure and the product is protected from external contamination.

In the US, NIOSH developed a draft protocol in 2015 to assess the performance of CSTDs in response to the increasing number of CSTDs in the marketplace and an awareness of scientifically non-robust and non-quantitative CSTD containment studies sponsored by commercial manufacturers [9]. The 2015 draft NIOSH protocol was designed to test the CSTDs’ effectiveness in containing drug vapor and preventing release into the surrounding environment within a chamber [9]. However, the protocol was not applicable to CSTDs that utilize air filtration technologies and employed a 70%:30% isopropyl alcohol (IPA):water mixture as the surrogate. IPA solvent is not representative of hazardous drugs because it generates an extremely high vapor concentration and it does not share physicochemical similarities with hazardous drugs [9]. The protocol measured transient IPA release during operation of the devices in a flow-through system and therefore was not able to provide a quantitative assessment of CSTD containment performance. In January 2016 NIOSH requested information to assist in development of a protocol to test air filtration CSTDs [10]. In September 2016, NIOSH released the 2016 draft NIOSH protocol for assessment of containment performance of all CSTD technologies [11]. The 2016 draft NIOSH protocol allows a quantitative assessment of all CSTDs irrespective of the technology that they employ, either physical barrier or air filtration [11].

The main difference with the 2016 draft NIOSH protocol is the measurement of released challenge agent within the chamber under non-flow-through conditions, which allows quantitative time weighted average (TWA) data to be obtained. The test requires the use of an appropriate semi-volatile challenge material from the list of nine agents proposed by NIOSH [11]. Air sampling methods form the basis of many standard analytical methods used by the United Kingdom (UK) Health and Safety Executive (HSE) and the US Occupational Safety and Health Administration (OSHA) for monitoring levels of volatile and semi-volatile materials in the environment [12].

Tandem air samples are collected on sorbent tubes placed within the NIOSH chamber. The air samples are analyzed using the gold standard analytical detection approach of automated thermal desorption gas chromatography mass spectrometry (ATD-GC-MS) and the released material quantified down to sub-ppb levels. Details are in accordance with the published HSE method MDHS104 [12].

The Biopharma Stability Testing Laboratory (BSTL) and the Health and Safety Laboratory (HSL) in the UK provided a response to the NIOSH request for information in January 2016 and this response is in excellent agreement with the latest draft NIOSH proposal [10,13].

The public comment period for the 2016 draft NIOSH protocol was closed on the 28^th^ of February 2018 [11]. One of the protocol’s current challenges is the lack of an established named surrogate that has been proven and can be used for assessment of CSTDs.

The present study provides a significant advancement toward a complete protocol by providing data for one surrogate, 2-phenoxyethanol (2-POE), one of nine potential candidate surrogates proposed by NIOSH in the draft protocol [11]. The OSHA Integrated Management Information System (IMIS) method P145 describes chemical sampling for 2-POE from the vapor employing air sampling method OSHA SLC1 [14,15]. The study analytical method is equivalent to the OSHA method and was validated using an approach based on OSHA T005 [16].

### USP800 requirements for a test protocol for CSTDs

With the launch of USP800, there is an urgent requirement for pharmacists to have access to scientifically robust test data that enable assessment of CSTD containment efficacy for the different available commercial CSTDs on the market to help inform a purchasing decision [17].

The 2016 draft NIOSH protocol builds on the earlier protocol and uses the same NIOSH chamber. [11]. BSTL has identified some important changes that should be made to the NIOSH chamber and its operation and these are detailed elsewhere and summarized in the S1 Text [18]. A significant revision to the 2016 draft protocol is the stipulation that all CSTDs must be used according to the manufacturer’s instructions for use (IFU), which was not included in the 2015 draft protocol. This led to some devices being compromised during testing.

### Challenge agent selection

The 2015 draft NIOSH protocol used a 70%:30% IPA:water mixture as the surrogate, which has since been rejected by NIOSH because it has a very high volatility and vapor breakthrough that is not representative of any of the classes of known hazardous drugs. The difference in behavior is due to the much lower vapor pressures of hazardous drugs [19,20]. The 2016 draft NIOSH protocol list of the nine candidates for use as potential challenge agents is shown in Table 1 (note IPA is included for comparison only). Table 2 shows the vapor pressures and Henry’s volatility constant values of a range of hazardous drugs for comparison.

**Table 1:** Nine potential challenge agents suggested by NIOSH in the draft protocol^a^ [11].

Compound	CAS^b^ number	Vapor pressure^c^ (mm Hg)	Henry’s constant H (atm m^3^/mole) Temperature = 298.15 K (25°C)
Dimethyl sulfoxide	67-68-5	0.61	1.51 × 10^−9^
Trimethyl phosphate	512-56-1	0.85	7.20 × 10^−9^
Tetramethylurea	632-22-4	1.16	8.48 × 10^−9^
Triacetin	102-76-1	0.002	1.23 × 10^−8^
Propylene glycol	57-55-6	0.129	1.28 × 10^−8^
Tetraethylurea	1187-03-7	0.208	2.80 × 10^−8^
Triethyl phosphate	78-40-0	0.393	3.60 × 10^−8^
2-Phenoxyethanol	122-99-6	0.007	4.72 × 10^−8^
Tripropyl phosphate	513-08-6	0.004	6.80 × 10^−7^
Isopropyl alcohol^d^	67-63-0	100	7.90 × 10^−6^

^a^Estimated values used where experimental data were unavailable generated using the Environmental Protection Agency (EPA) EPI SUITE software [21].

^b^Chemical Abstracts Service Registry.

^c^Vapor pressure refers to the active pharmaceutical or active ingredient in its pure state.

^d^ Vapour pressure data of Isopropyl alcohol shown for comparison purposes only.

**Table 2:** Physicochemical data for some commonly used hazardous drugs.

Compound	CAS^a^ Number	Vapor pressure (mm Hg)	Henry’s Constant H (atm m^3^/mole) Temperature = 298.15 K (25°C)
Cisplatin	015663-27-1	0.0000135^b^ 4.82 × 10^−20^^c^	2.091 × 10^−26^
Carboplatin	041575-94-4	1.33 × 10^−9^^c^	5.51 × 10^−14^
Thiotepa	52-24-4	0.00941^c^	2.83 × 10^−10^
Carmustine	154-93-8	0.000142^b^ 0.000293^c^	4.76 × 10^−11^
Cyclophosphamide	50-18-0	0.0000247^b^ 4.4 × 10^−5^^c^	1.36 × 10^−11^
Etoposide	33419-42-0	0.0000195^b^ 8.98 × 10^−22^^c^	1.75 × 10^−30^
5-Fluorouracil	51-21-8	0.0000105^b^ 7.12 × 10^−8^^c^	1.66 × 10^−10^

^a^Chemical Abstracts Service Registry.

^b^(Kiffmeyer *et al*., 2002) [20].

^c^Calculated using the modified grain method using Environmental Protection Agency (EPA) EPI SUITE software [21].

Parenteral solutions of hazardous drugs are either supplied as lyophilized powder for reconstitution or as a ready-to-administer solution. Administration is performed using an aqueous solution, which means the amount of drug present in the vapor above the liquid is estimated using Henry’s volatility constant and not by the vapor pressure of the pure substance. The Henry’s volatility constant is able to predict the chemical vapor concentration resulting from liquid leakage of hazardous drugs. We have selected 2-POE as the surrogate of choice based on its physicochemical properties, including its Henry’s constant, which is more than two orders of magnitude greater than that of the most volatile hazardous drug, 5-Fluorouracil (when presented as an aqueous solution). Using a 2.5% v/v solution of 2-POE, a liquid release volume as low as 1 μL can be detected using the analytical methods described in this study, which is due to the high sensitivity of detection, less than 1 ppb of release within the chamber. Smaller volumes of 2-POE release in the liquid state will not be detected due to the experimental limit of detection (LOD) and limit of quantitation (LOQ) demonstrated.

Vapor pressures of pure active pharmaceutical ingredients as determined by Kiffmeyer *et al* [20] are not relevant to the study of pharmaceutical drugs which are formulated products and not pure chemical substances [19,20]. Hazardous drug materials in an aqueous solution inside a drug vial typically have vapor concentrations that are 4–24 orders of magnitude lower than that generated by 70% IPA based on their relative Henry’s volatility constant values.

BSTL and HSL proposed the use of 2.5% v/v 2-POE in water as challenge agent to be used within the 2016 draft NIOSH protocol, the response can be found on the docket [13]. 2-POE is more representative of real hazardous drugs and has sufficient solubility in water to generate a clinically relevant drug concentration of ~25 mg/mL [18]. The saturated vapor pressure of 2-POE (1.3 Pa) is over 1000 times less than that of IPA (4400 Pa), but is over 100 times greater than those of hazardous drugs at 20°C [20]. When 2-POE is reconstituted, its chemical vapor concentration is at least two orders of magnitude higher based on the Henry’s volatility constant data than Thiotepa or 5-Fluorouracil. An additional important characteristic of the surrogate, when considering its use in the NIOSH protocol, is that its aqueous solution generates sufficient vapor concentration to be easily detectable using the analytical methods employed in the protocol. Extremely low vapor concentrations make it impossible for hazardous drugs to be tested in the NIOSH protocol using detection by ATD-GC-MS.

## Materials

2.5% v/v 2-POE was prepared by addition of 2.5 mL of 2-POE (Sigma Aldrich, 90% or better, CAS No. 122-99-6) to 97.5 mL of Milli-Q water (Integral system, Merck Millipore, UK), mixed by shaking, and 50 mL aliquoted into 100 mL type I glass vials (Adelphi, UK). 100 mL drug vials (Adelphi, UK) were prepared by adding 50 mL of Milli-Q water, and sealed. The Milli-Q-containing vials were used for all negative control experiments. Thermal desorption (TD) tubes 89 mm long × 6.4 mm outer diameter and packed with 200 mg of Tenax TA (Markes, UK) were used. Internal standard was prepared by dissolving d8 toluene (Sigma Aldrich, UK) in analytical grade methanol (Sigma Aldrich, UK).

## Equipment

A NIOSH chamber was constructed as described in the 2015 draft NIOSH protocol with specific modifications described by Wilkinson *et al*. [18]. The chamber was sealed during testing using hand spring clamps applied to the flexible tubing (Tool station, UK). Three calibrated air sampling pumps (Gil-Air, UK) were used, two to sample the chamber air for both blank and test measurements and one to collect the environmental background air on each day of test. TD tubes were conditioned using a Markes TC-20 prior to use. A standard atmosphere equipment (HSL, UK) as described in HSE method MDHS104 [12] was used to load d8 toluene (Sigma Aldrich, UK) internal standard to TD tubes. An external B105 diaphragm pump (Charles Austin, UK) was used to purge the chamber with clean air prior to use. Sample analysis was performed using an automated PerkinElmer TD-650, a PerkinElmer Autosystem GC interfaced to a PerkinElmer TurboMass MS system, a VOCOL capillary column (60 m length × 0.25 mm diameter × 1 μm film thickness) and PC data collection and analysis software supplied by PerkinElmer (TurboMass V5.4.2.1617; 2008).

## Methods

For additional detail, please refer to the supplemental materials provided electronically.

The assessment of CSTD containment performance was performed in accordance with the 2016 draft NIOSH protocol (11) as follows:

- A 2.5% v/v POE solution was used as the challenge agent
- An air purge of the chamber was performed at a flow rate of 15 L/min for 30 minutes prior to use
- An environmental air sample was taken each day prior to testing
- Air samples were collected at a flow rate of 100 mL/min and for 30 minutes using dual air sampling pump systems connected to Tenax TA TD tubes
- Dual blank chamber measurements were recorded prior to testing of a CSTD to ensure no contamination inside the chamber
- A robust chamber cleaning procedure was established and used throughout the testing

In the 2016 draft NIOSH protocol, the air sampling is stopped after completion of the NIOSH tasks 1 and 2. However, in the present protocol the pumps continue collecting air for a fixed duration of 30 minutes. NIOSH tasks 1 and 2 were performed as stated in the 2016 draft NIOSH protocol except that the number of tests was increased to five [11]. Negative and positive controls were performed. A positive control (“open system”: manipulations of 2.5% POE according to each of tasks 1 and 2 using a standard needle and syringe) was used for comparison with test samples to provide a reference measurement for the maximum level of vapor release when performing tasks 1 and 2 without a CSTD. Negative controls involved performing the same tests but using 100% Milli-Q water as the challenge agent and verified that the source of POE release was from the challenge agent used in the test and not from any of the equipment used in the testing.

### Sample analysis

**Fig 1:**
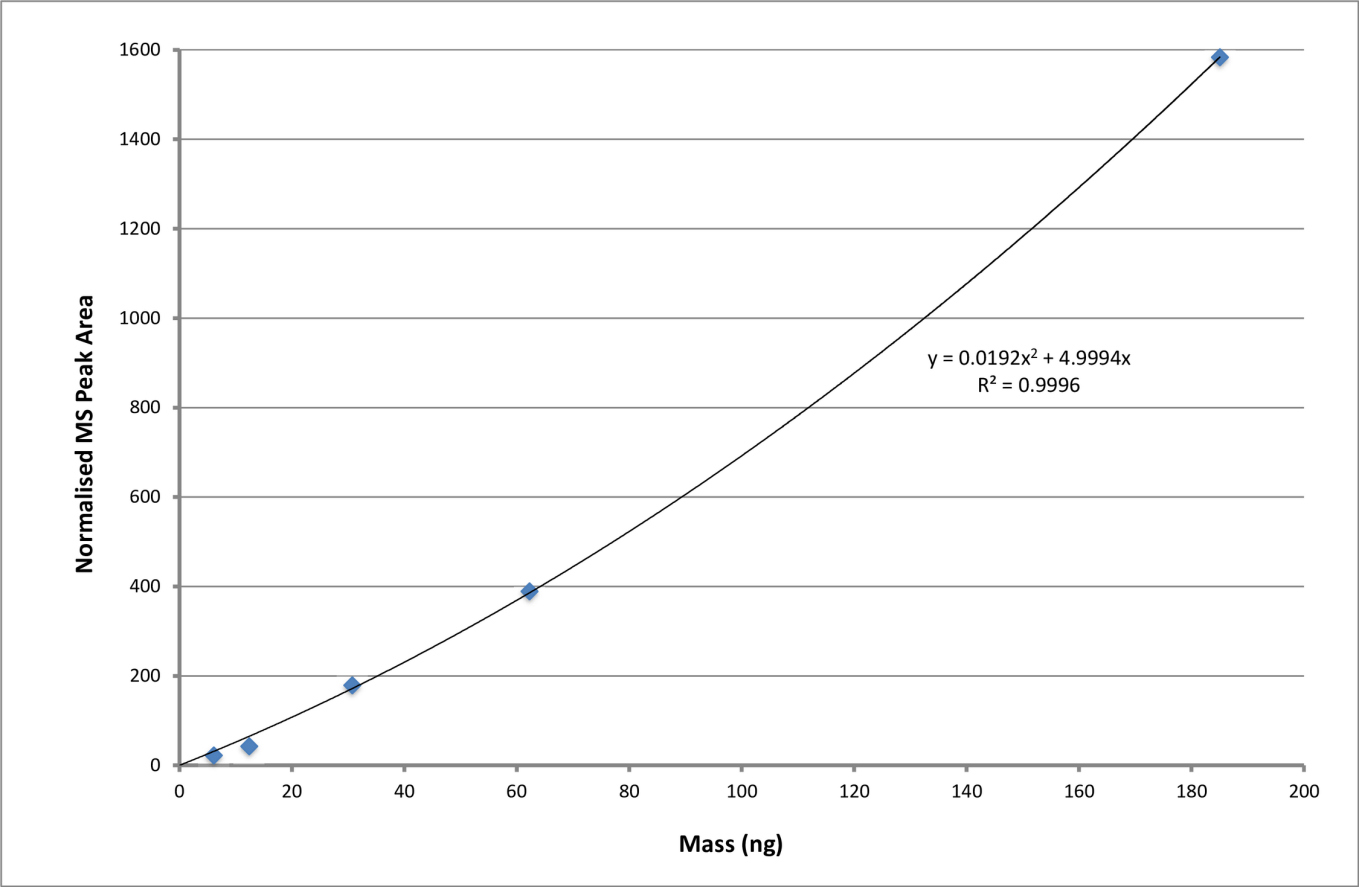
Calibration plot for 2-phenoxyethanol by automated thermal desorption gas chromatography mass spectrometry.

Sorbent tubes were analyzed using automated ATD-GC-MS detection. Analytical conditions and instrument calibration are described fully in the supplemental materials. A TWA sample approach allowed for determination of the mass of POE collected on the sorbent tubes, in nanograms, using a calibration curve of POE (Fig 1). The mass of POE was then converted to an airborne concentration in the test chamber in ppb following the procedures stated in HSE MHDS104 [12].

**Fig 2:**
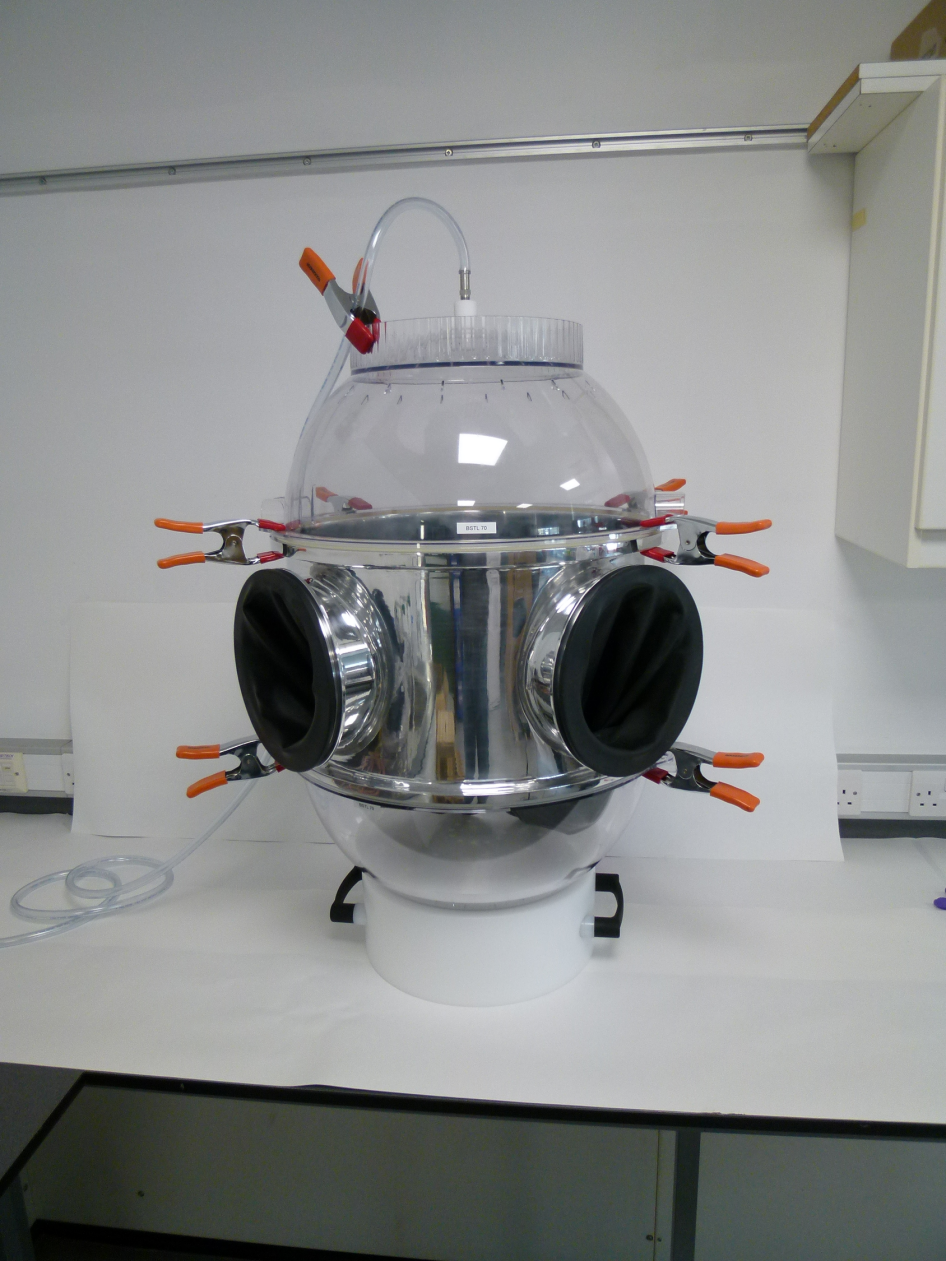
Biopharma Stability Testing Laboratory modified National Institute for Occupational Safety and Health test chamber with aluminum extension piece.

Data processing, determination of the experimental LOD and LOQ, and data analysis were performed in accordance with the HSE MHDS104 method [12]. The POE release values are reported without background subtraction of the blank samples recorded immediately prior to the test. Tests in which carryover in the chamber was observed from a previous test, where a high release of POE was observed, were repeated following a further cleaning of the chamber. The implementation of a robust cleaning procedure ensured that all POE residues were removed prior to test. This also helped to ensure that the low LOD was maintained throughout the testing. Fig 2 shows the BSTL modified NIOSH chamber. All changes to the original NIOSH chamber are documented and described elsewhere [18].

## Results

The breakthrough tests performed using sample volumes of 3, 6, and 12 liters confirmed that there was no breakthrough using the air sampling approach described, and furthermore that >99.9% recovery was achieved from the Tenax TA air sampling tubes, meaning that 2-POE is totally desorbed by the analytical procedure. Under the conditions of testing this is well within the upper limits for breakthrough for the Tenax sorbent media. We also demonstrate that no carryover was observed, *i*.*e*. that no 2-POE was detected on any of the system blank tubes analyzed after each test sample. Using the analytical method described, an instrumental LOD of less than 0.1 ng for 2-POE and an experimental LOD of 0.4 ppb were demonstrated.

A total of four commercially available CSTDs that employed either physical barrier (PhaSeal, Equashield, and ChemoClave) or air filtration technology (Tevadaptor, marketed as OnGuard in the US) were included in our study (Table 3).

**Table 3:** CSTD containment performance data using 2.5% v/v 2-phenoxyethanol (2-POE) as challenge agent according to draft NIOSH protocol and defined tasks: task 1 (n = 5) and task 2 (n = 5) performed under IFU conditions.

CSTD product	NIOSH task	Mean concentration of 2-POE (ppb) ± (95% CI)
Tevadaptor (marketed as OnGuard In USA, Teva)	1	≤ 0.88*
	2	≤ 0.88*
PhaSeal (BD)	1	≤ 0.88*
	2	≤ 0.88*
ChemoClave vial shield and Spiros (ICU Medical)	1	2.90 ± 2.25†
	2	7.50 ± 17.86†
Equashield (Equashield)	1	≤ 0.88*
	2	≤ 0.88*
Needle and syringe	1	4.20 ± 2.23†
	2	5.10 ± 1.71†
Blank (N = 85)	—	0.12 ± 0.02†

CSTD: closed system transfer device; IFU: instructions for use; NIOSH: National Institute for Occupational Safety and Health; ppb: parts per billion.

* Limit of detection was determined to be 0.35 ppb and the limit of quantitation was determined to be 0.88 ppb.

† range calculated at the 95% confidence interval (CI).

A larger number of replicates were used in this study (n = 5) than recommended in the original protocol (n = 4) [9,18]. With five replicate tests and duplicate air sampling for each test, a total of 10 vapor measurement values were obtained for each CSTD to allow basic statistical analyses and examination of containment efficacy.

**Fig 3:**
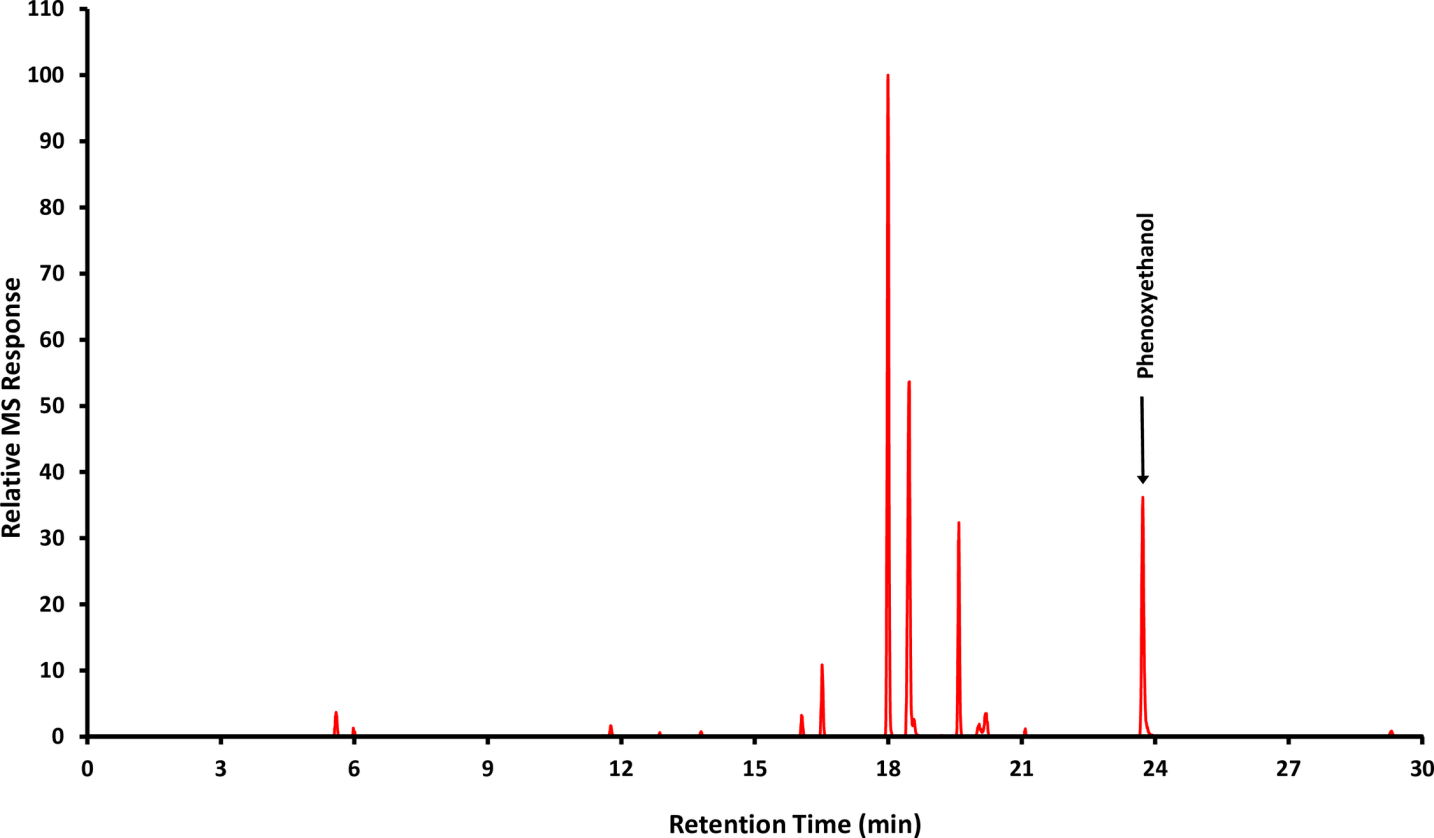
Typical gas chromatogram for 2-phenoxyethanol with mass spectrometry (selected M/Z values: 77, 94, and 138) detection.

Chamber blank measurements (n = 85) and positive controls (“open system” with needle and syringe, n = 2) were performed prior to each device test as described in the modified NIOSH protocol. No background contamination was present (data not shown). Additional blank measurements were performed using water as a negative control (n = 2). A typical gas chromatogram signature for 2-POE detection by mass spectrometry is shown in Fig 3.

Results for 2-POE concentration in the test chamber for tasks 1 and 2 are presented in Table 3. The average positive control value (n = 2) using needle and syringe was 4.20 ± 2.23 ppb for task 1 and 5.10 ± 1.71 ppb for task 2 at the 95% CI. The average blank control value (n = 85) was 0.12 ± 0.16 ppb at the 95% CI and was used to estimate the LOD and lower limit of quantitation (LLOQ) according to equations 3 and 4 of the modified protocol [11].

**Fig 4:**
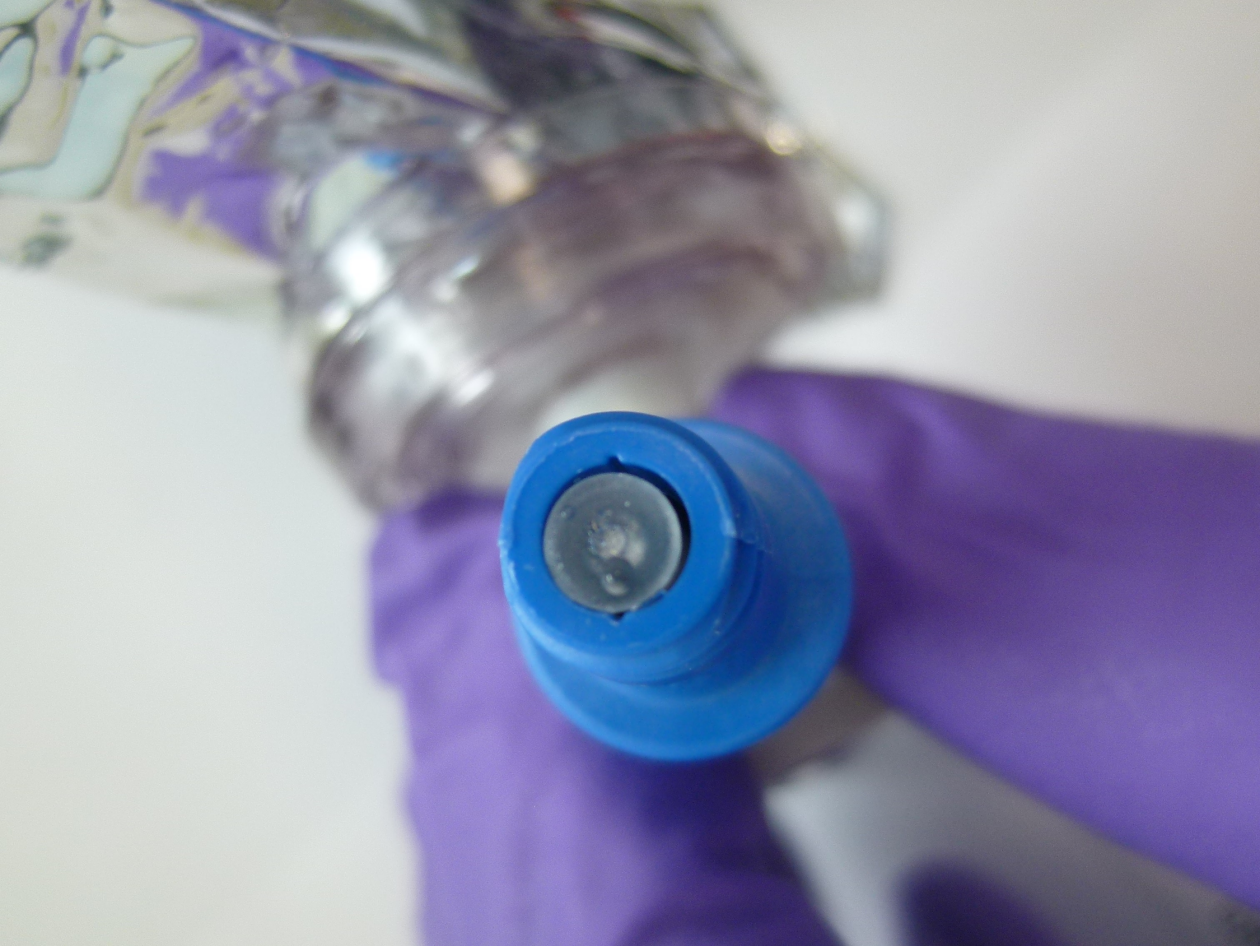
Liquid droplets observed on the membrane of ChemoClave CSTD following task 2.

The average values obtained for PhaSeal, Equashield, and Tevadaptor (n = 5 each) were below the blank control-corrected LLOQ for each of the two tasks (≤ 0.88 ppb). However, ChemoClave’s Vial Shield with Spinning Spiros produced values that were 5 to 25 times the LOD, and one measurement was 120 times higher than the LOD. The higher values obtained with the ChemoClave device can be attributed to the liquid residue observed on the membranes following disconnection (Fig 4), resulting in high vapor release during CSTD manipulation [18].

A strength of the proposed protocol when 2-POE is employed as challenge agent is that devices that produce a clearly observable liquid release give much higher vapor concentrations of 2-POE. This is because the 2-POE released is quickly taken in to the vapor and measured. In contrast, devices that did not appear to produce any liquid release gave either below LOQ or close to LOQ values for 2-POE. The findings suggest that the protocol provides a means to differentiate devices that give a release of liquid from those that do not, and that vapor release is much less significant compared with liquid release in terms of increasing the vapor concentration of 2-POE.

## Discussion

An independent evaluation and review of the first draft NIOSH protocol from 2015 identified several limitations [18]. First, the protocol was designed or intended for physical barrier CSTD technology only and therefore was not appropriate for use with air filtration devices, according to NIOSH [18]. IPA has a vapor pressure that is five to six orders of magnitude higher than most hazardous drugs and efficiency of capture by charcoal filter devices is suboptimal. The use of 70% IPA also contributed to raising the vapor concentration above the optimum threshold for capture based on charcoal filter devices and is not representative of drug solution concentrations. The selection of challenge agent in the 2015 protocol prevented the hospital pharmacist from evaluating all CSTDs on the market. Second, a 70% IPA/water solution was not representative of hazardous drugs in terms of chemical structure and vapor pressure, and did not reflect the concentrations of drugs handled in the clinic [18]. Third, the 2015 draft NIOSH protocol did not provide quantitation of the total vapor released from the CSTD, measuring transient IPA release values only [18]. Fourth, the NIOSH statement for using the CSTDs in the protocol compromised the operation of some CSTDs during the test. Only CSTDs requiring injection of air prior to withdrawal from the vial were totally compatible with the protocol. Incorrect IFU resulted in false positive results for CSTD technologies that were incompatible with air injection, invalidating the results obtained [18].

There are other practical concerns associated with the detection devices used in the 2015 draft NIOSH protocol design [9]. Real-time detection devices, such as the filter-based MIRAN infrared instrument used in that protocol, do not provide real-time detection of vapors. The MIRAN detector did not sample directly from the environment; instead the sample was introduced to the detector from the NIOSH chamber by an interconnecting length of flexible tubing. Under the conditions of the draft NIOSH protocol, the IPA challenge agent would need to be transported from the point of release in the chamber to the gas flow detection cell inside the MIRAN instrument. The time for this journey is dependent on the gas flow rate, but is estimated to be several minutes even with an optimistic flow rate of 10 L/min. This flow rate is beyond the capability of MIRAN’s internal pump when operating in a restricted gas flow, *i*.*e*. under the conditions ofthe 2015 draft NIOSH protocol [9]. Furthermore, IPA is continually being mixed with additional air that is entering the chamber as it travels from the chamber to the MIRAN detector. This dilution effect changes the IPA concentration in an unpredictable way and prevents a robust quantitation of the release values. The high IPA composition of 70% used in the 2015 draft NIOSH protocol was necessary to compensate for the MIRAN’s low detection sensitivity of parts per million [9,13]. However, we observed a number of issues affecting the CSTD equipment when using 70% IPA; for example, condensation in parts of the physical barrier balloons that should not contain liquid. This impaired the operation of the CSTD equipment, further supporting the need for a more suitable challenge agent.

The TWA approach proposed in the current 2016 draft NIOSH universal protocol does provide a truly quantitative assessment of containment performance of CSTDs [11,13]. By sampling the amount of challenge agent in the sealed chamber with known volume over time, it is possible to directly relate the amount captured on the sampling tube to the total amount of release in the chamber. A simple mathematical adjustment or scaling factor is needed to scale from the 3 liters of air collected on the tubes to the total volume of the chamber, which was determined to be 127 liters. The values obtained represent the total amount of 2-POE released from the closed system through a combination of aerosol, vapor, and liquid release. The details for how the system was verified are provided in the S1 Text. In short, this was achieved by aliquoting known amounts of liquid into the chamber on an inert solid support. The system was verified up to 50 μL of release only, but above this amount of leakage the vapor concentration of 2-POE was significantly higher than the open system of needle and syringe. It should be noted that with very high liquid releases above 50 μL not all of the liquid may be evaporated fully and the measured vapor concentration may be an underestimate. With liquid releases between zero and 50 μL there is sufficient evaporation to ensure that all the available 2-POE is transferred to the vapor during the test.

The unified 2016 draft NIOSH protocol employing ATD-GC-MS analysis allows both air filtration and physical barrier CSTDs to be assessed and represents a paradigm shift from the pre-NIOSH protocol and the 2015 draft NIOSH protocol era, when it was not possible to compare CSTDs based on robust scientific data [11,13]. With the current collaborative BSTL and HSL implementation of the protocol it is possible to assess the containment performance of different CSTD technologies and compare them in a head-to-head test [13]. ATD-GC-MS has been the gold standard for a number of standard analytical methods published by the International Organization for Standardization [22], the Environmental Protection Agency, and OSHA for detecting vapors from volatile and semi-volatile chemicals. ATD-GC-MS detection, with sub-ppb detection capability, is more than 1000 times more sensitive than the MIRAN equipment used in the 2015 NIOSH protocol and provides very high selectivity as well as sensitivity [18].

The change in challenge agent from IPA to 2-POE presents a more representative surrogate for hazardous drugs in terms of vapor pressure, Henry’s volatility constant, chemical structure, and physicochemical behavior. Furthermore, 2-POE can be efficiently cleansed from the testing chamber following a high-level release using a validated cleaning protocol, enabling CSTD testing to continue without delays. In contrast, potential challenge agents that are much more persistent and difficult to remove from the test chamber following a high release are impractical to use. Recent published work by Wilkinson *et al* demonstrates the difficulties in cleaning the NIOSH chamber in between releases when TEU is used as challenge agent in the NIOSH testing [23]. In addition, operating CSTDs according to the manufacturers’ IFUs reduces potential confounding factors and artifacts when performing the containment assessments.

The study data obtained using our modified, universal protocol showed that CSTDs with both air filtration and physical barrier technology can prevent the release of hazardous drugs as vapor, liquid, and liquid-based aerosols during compounding and administration tasks performed in the clinic. Our data also demonstrated that Tevadaptor (OnGuard), which employs air filtration technology, produced containment efficacies equivalent to the two physical barrier CSTDs tested, including the reference predicate product PhaSeal [18].

There are additional challenges that NIOSH may address in relation to CSTD assessments. As no acceptance criteria have been suggested by NIOSH, it is currently impossible for hospital pharmacists to make an informed purchasing decision based on the release data from the protocol. We recommend that acceptance criteria should be established based on either providing a protection factor (PF) over that of using an open system of needle and syringe, or on the experimental LOQ value of the CSTD when challenged. In this study we report efficacies of CSTDs based on the release values being above or below LOQ using the assay described, which has sub-1 ppb sensitivity. A CSTD that has containment performance yielding below-LOQ values represents the highest efficacy that can be obtained. Other air sampling approaches could be adopted as an alternative to ATD-GC-MS; for example, diffusive sampling using solid phase microextraction or sorbent pen, and active sampling using a needle trap. However, investigations performed by BSTL and HSL using alternative sampling approaches did not show potential for use under the NIOSH protocol due to lack of sensitivity in detection for 2-POE. As such, the ATD-GC-MS was shown to be the most sensitive air sampling detection strategy.

## Conclusions

2-POE has proved to be an ideal surrogate for testing of CSTDs using the NIOSH protocol. Equashield, Tevadaptor (OnGuard), and PhaSeal all produced release values that were below the LOQ for the assay, whereas the ChemoClave Vial Shield with Spinning Spiros system released higher amounts than the open system of needle and syringe, showing that physical barrier as well as air filtration technology can provide hazardous drug containment.

We propose that acceptance criteria be established for scoring the containment performance of CSTDs either based on the experimental LOQ or a PF approach where: 1.) a PF must be significantly higher than the proportion between the values obtained for the open system divided by the experimental LOQ of that system and 2.) the experimental LOQ must not be greater than 1 ppb.

In our system, Equashield, Tevadaptor (OnGuard), and PhaSeal provided a PF greater than 5.8 for task 1 (4.1 ppb/0.71 ppb) and a PF greater than 7.2 for task 2 (5.13/0.71) and therefore these systems meet the acceptance criteria for significantly reducing operator exposure and are qualified for handling hazardous drugs.

CSTDs that do not provide any additional protection to the operator over an open system when handling hazardous drugs or even release higher amounts of hazardous drugs, cannot be considered a form of protective equipment, and these devices are therefore unsuitable for use with hazardous drugs.

## Supporting information

S1 Text Supplementary materials. (DOCX)
